# If I Know Myself, I Can Welcome You: Identity Roots of Intergroup Solidarity

**DOI:** 10.1002/jad.70154

**Published:** 2026-04-12

**Authors:** Fabio Maratia, Elisabetta Crocetti

**Affiliations:** ^1^ Department of Psychology Alma Mater Studiorum University of Bologna Bologna Italy

**Keywords:** Adolescence, identity statuses, integration policies, intergroup solidarity, societal well‐being

## Abstract

**Introduction:**

While implementing integration policies is crucial for countries to foster cohesion and well‐being, it is equally important to understand how individuals, especially youth, endorse such policies and the factors that influence this form of intergroup solidarity. Adolescents' identity statuses can influence various individual and interpersonal outcomes, but little is known about their relation with societal outcomes. Schools are crucial contexts where adolescents develop their educational identity, which is influenced by characteristics such as their ethnic background and, in turn, can influence how youth perceive the diversity around them. This study examined whether youth intergroup solidarity (i.e., positive attitudes toward migrant integration policies) differed over time based on their educational identity statuses (i.e., achievement, early closure, moratorium, searching moratorium, and diffusion) and if these differences are replicated among native Italian and migrant adolescents.

**Methods:**

A total of 1547 adolescents (*M*_age_ = 15.69 years, SD_age_ = 1.22, 52.96% male, 79.94% native Italians) participated in a 3‐year longitudinal study with seven assessments.

**Results:**

Adolescents in the achievement status of their educational identity exhibited the highest level of intergroup solidarity; adolescents in the early closure, moratorium, and searching moratorium statuses reported intermediate levels; and adolescents in the diffusion status reported the lowest level of intergroup solidarity. These results were confirmed among native Italian and migrant adolescents.

**Conclusions:**

This study highlighted that youth intergroup solidarity is associated with adolescents' educational identity. Promoting a stable identity in this domain could foster greater support for integration policies, ultimately facilitating their implementation and enhancing their benefits for social cohesion and well‐being.

1

In increasingly divided societies, marked by economic, political, and social upheavals between individuals with different ethnic and cultural backgrounds (United Nations 2020), it is essential for countries to promote migrant integration policies, not only to level these differences (Solano and Huddleston 2020) but also to promote greater solidarity between citizens (Berry 2011) and enhance their well‐being (Sand and Gruber 2018; Tatarko et al. 2021). Furthermore, since the implementation of these policies is intertwined with public opinion (for a review, see Baum and Potter 2008), it is important to understand how much individuals endorse such policies and what factors may influence this form of intergroup solidarity. Understanding the development of intergroup solidarity is especially crucial during adolescence, when young people face the dual challenge of developing social perspectives (Albarello et al. 2020) while forming their own identity (Branje et al. 2021), as they become increasingly aware of social groups as well as their own sense of self and how it relates to others. Adolescents cope with these two tasks in various ways, and as a result, they may, on the one hand, develop more positive or negative views toward other social groups (Verkuyten 2022), and, on the other hand, reach different identity statuses (Marcia 1966; Meeus 2011). Yet, are these two key developmental endeavours distinct, or are they intertwined? A large corpus of research shows that identity statuses are characterized by distinct profiles, especially regarding psychosocial adjustment indicators and the quality of family and peer relationships (for a review, see Crocetti 2018). In contrast, less is known about how adolescents in various identity statuses differ in their intergroup perspectives, with most evidence focusing on other social outcomes, such as, for instance, youth levels of social responsibility (Crocetti et al. 2012).

Within this framework, a crucial identity domain for developing social orientations may be the educational one. Indeed, school represents a key developmental and acculturative context (Schachner et al. 2018), where adolescents are first exposed to social diversity (Maratia et al. 2025), begin to interact with peers from different backgrounds, and start forming their own ideas about social groups (Verkuyten 2022), while engaging in identity tasks (Negru‐Subtirica et al. 2023). Moreover, school plays a central role in transmitting civic values that are likely to shape adolescents' social perspectives (Blasko et al. 2018). Through both formal education, such as civic and citizenship education programs, and informal socialization processes within the school context, adolescents learn principles of equality, justice, and respect for diversity (Luken Raz and Killen 2024). Thus, the way adolescents develop their educational identity may shape how they engage with and internalize the social experiences and values encountered at school. In this context, identity processes such as commitment, in‐depth exploration, and reconsideration reflect the extent to which adolescents invest in, reflect upon, and evaluate the role of education in their lives (Negru‐Subtirica et al. 2023). These processes combine into distinct identity statuses that represent relatively stable configurations of adolescents' orientation toward education. Adolescents in identity statuses characterized by stronger commitment and active exploration of the meaning and importance of what they learn at school may be more likely to integrate the civic and social values encountered in this context. Conversely, adolescents in identity statuses characterized by weak commitment or high reconsideration may be less engaged with the educational domain and therefore less likely to internalize these orientations. Consistent with this reasoning, previous research has shown that specific processes related to educational identity, such as the educational in‐depth exploration, are associated with fewer negative attitudes toward members of ethnic minority groups among majority youth (Bobba et al. 2023), suggesting that educational identity statuses characterized by a deeper engagement with the educational domain may be linked to more inclusive intergroup orientations and, consequently, greater solidarity. In light of this, the current study aimed to examine whether adolescents' intergroup solidarity (i.e., positive attitudes toward migrant integration policies) differed over time based on their educational identity statuses (i.e., achievement, early closure, moratorium, searching moratorium, and diffusion), derived from the combination of different identity processes (i.e., commitment, in‐depth exploration, and reconsideration of commitment) assessed at the beginning of the study. Furthermore, it was assessed if these differences were replicated for native Italian (i.e., adolescents and their parents were born in Italy) and migrant (i.e., youth or at least one of their parents was born outside Italy) adolescents.

## 2 Integration Policies, Intergroup Solidarity, and Well‐Being

Modern societies are characterized by the interaction and coexistence of individuals with and without a migrant background (European Commission 2023). Focusing on the integration process and the factors that can facilitate it is essential to enhance solidarity between these two groups. Integration can be facilitated when governments implement policies that foster equal rights, opportunities, and security for migrants (Solano and Huddleston 2020). These policies not only facilitate migrants' integration but also promote greater well‐being (Sand and Gruber 2018; Tatarko et al. 2021) and social cohesion (Green et al. 2020), enhancing individuals' intergroup solidarity rather than threat (Schlueter et al. 2020) and promoting more positive interethnic attitudes (Kim and Byun 2019) and greater support for migrants' rights (Barber et al. 2013) among youth.

Given their importance, tools like the Migrant Integration Policy Index (Solano and Huddleston 2020) monitor to what extent countries implement policies promoting the integration of migrants. However, these policies do not operate in a vacuum (Maratia et al. 2026); rather, they are intertwined with public opinion (for a review, see Baum and Potter 2008), which can significantly influence their implementation and effectiveness. In this regard, it is essential to acknowledge that such policies do not always produce favourable outcomes, nor are they always positively received by citizens; instead, they may elicit feelings of anger, envy, or resentment. Indeed, evidence shows that integration policies, especially when they are perceived as exclusionary, that is, benefiting only specific groups (Callens 2015), or when they are seen as less clear and coherent (De La Sablonnière et al. 2020), can have unintended effects, such as increased perceptions of threat from immigrants, stronger anti‐immigrant attitudes, and even negative repercussions for psychological well‐being. For all these reasons, it becomes essential to understand the extent to which individuals, especially youth, endorse these policies and which factors can influence this endorsement, thus enhancing their positive outcome in terms of intergroup solidarity.

### 2.1 Intergroup Solidarity during Adolescence

During adolescence, young people define their societal attitudes and how they relate to different social groups, thus making this period critical for forming and consolidating views toward the world around them (Eger et al. 2022). This developmental stage is therefore pivotal for developing intergroup orientations, such as those related to integration policies, that will inform their future adult perspectives (Rekker et al. 2015). Such orientations may eventually play a role in shaping national and local integration policies, considering that policy development and public opinion are closely interconnected (for a review, see Baum and Potter 2008). Notably, during this period, the likelihood of showing more or less intergroup solidarity may depend on the specific personal characteristics of adolescents. For instance, research has shown that sex differences matter, with females generally expressing stronger solidarity and more positive attitudes toward minority groups than males (e.g., Maratia et al. 2024). Alongside sex, age can play a dynamic role in distinguishing between more or less inclusive youth. As adolescents mature, enhanced cognitive skills and moral reasoning can support the development of empathy and inclusiveness (Bayram Özdemir et al. 2021), thus fostering more intergroup solidarity. However, this developmental trajectory is not uniform, as growing social awareness may also lead some adolescents to develop decreased social trust, particularly when they perceive outgroups as potential threats to their future socio‐economic opportunities (for a meta‐analysis on the development of prejudice in adolescence, see Crocetti et al. 2021). Socio‐economic background further contributes to these differences, as adolescents from higher‐status families (e.g., parents with a high educational level) tend to report more favourable attitudes toward ethnic minorities (Kim and Byun 2019). Thus, focusing on the factors that drive adolescents' formation of inclusive perspectives can help understand the development of intergroup solidarity among youth and shed light on what could facilitate the implementation of migrant integration policies, enhancing their benefits for social cohesion and well‐being.

### 2.2 The Impact of Identity Formation on Individuals and Society

Building upon Erikson's theorization (1950, 1968), according to which identity formation is the most important developmental task for adolescents, different models have been proposed to understand how youth cope with this task (e.g., Berzonsky 1989; Marcia 1966; Schwartz 2001). In this regard, the identity status paradigm (Marcia 1966, 2001) conceptualized specific identity statuses based on the extent to which young people have resolved or not the identity crisis by assuming commitments after an eventual period of active exploration. Along this line, process‐oriented models have been introduced to expand the identity status paradigm and capture how identity is formed, changed, and consolidated dynamically over time (Crocetti et al. 2023; Luyckx et al. 2023; Meeus 2011).

Within this framework, the three‐factor identity model focuses on three pivotal identity processes (Crocetti et al. 2008). The commitment process indicates a feeling of certainty about chosen goals and values, which is often associated with stability and security. In‐depth exploration involves an active evaluation and reflection on one's current commitments. It can be a double‐edged sword, as it may lead either to the verification and consolidation of current commitments or to their reconsideration, an identity crisis that implies a potential change in actual commitments after comparing them to alternative options. These processes are at the basis of two iterative cycles, such as identity formation (i.e., based on the interplay between commitment and reconsideration of commitment) and identity maintenance (i.e., based on the interplay between commitment and in‐depth exploration). While identity formation involves revising commitments based on appealing alternatives, identity maintenance involves examining and validating current commitments, eventually returning to the previous cycle if they no longer align with one's sense of self (Meeus 2018).

Based on specific combinations of these three processes, adolescents can be in five different identity statuses (Crocetti et al. 2008). The **achievement status** consists of individuals scoring high on commitment and in‐depth exploration but low on reconsideration of commitment. The **early closure status** reflects a moderately high commitment with low in‐depth exploration and reconsideration of commitment. The **moratorium status** is characterized by low commitment and low in‐depth exploration but high reconsideration of commitments, while the **searching moratorium status** combines high levels of all the processes. Finally, the **diffusion status** portrays low levels of commitment, in‐depth exploration, and reconsideration of commitment (Meeus et al. 2012). Prior longitudinal research shows that a large share of adolescents (63%) remain in the same identity status over a 5‐year period, whereas those who do change tend to show either progressive or regressive transitions (Meeus et al. 2010).

From this perspective, considering adolescents' identity status at a given time point is particularly informative, as extensive research shows that different identity statuses are associated with distinct psychosocial profiles. In this vein, most research focuses on correlates at the individual (e.g., well‐being; Morsunbul et al. 2016) and interpersonal (e.g., relationship with family members; Albert Sznitman et al. 2019) levels. Specifically, regarding individual outcomes, adolescents in the achievement status, followed by those in early closure, generally show more psychosocial adaptive patterns (Schwartz et al. 2011), including lower levels of loneliness and depressive symptoms (e.g., Park et al. 2023; Raemen et al. 2022), than adolescents in searching moratorium, moratorium, and diffusion. Regarding interpersonal relationships, adolescents in identity statuses characterized by a high degree of commitment (i.e., achievement and early closure) report better communication quality with their mothers (Crocetti et al. 2008) and a more positive family climate (Albert Sznitman et al. 2019). Thus, displaying a more stable identity is associated with positive individual and relational outcomes.

In contrast, the societal and collective aspects intertwined with specific identity statuses have received less attention so far (for a review, see Crocetti 2018). Most studies exploring these associations focus on the likelihood that identity formation is linked with adolescents' social participation. In this vein, adolescents in the achievement status report higher scores of values‐oriented volunteer functions (Marinica and Negru‐Subtirica 2020) and social responsibility (Crocetti et al. 2012), demonstrating a stronger aspiration to contribute to their communities. Conversely, adolescents in the diffusion status report low commitment to civic activities (Crocetti et al. 2012).

The positive outcomes linked to more stable identity statuses may not only help adolescents feel more integrated within the social fabric (Chan and Mak 2020), but can also indirectly foster the development of intergroup solidarity and social cohesion (e.g., Davies et al. 2024; Gagliardi et al. 2024). This may happen because factors such as volunteering, civic responsibility, and social engagement can support a deeper understanding of the significance, for oneself and others, of important social, civic, political and humanistic values (Belyaeva 2024; Procentese and Gatti 2022), and promoting more positive and meaningful relationships with others (e.g., Aked 2015), regardless their ethnic and cultural background. However, there is still a dearth of research on the direct link between identity statuses and intergroup solidarity. Furthermore, in line with previous research emphasizing the importance of examining specific identity domains to gain a more nuanced understanding of their implications for adolescents' psychosocial development, it is reasonable to focus on educational identity (Negru‐Subtirica 2024). This core domain can significantly influence how adolescents engage with civic, social, and political values (Luken Raz and Killen 2024), as well as how they learn to navigate the high levels of ethnic and cultural diversity they encounter across multiple areas of life.

### 2.3 Educational Identity and Intergroup Solidarity

Education represents the primary and normative life domain for adolescents during this life stage (Negru‐Subtirica et al. 2017), and their educational identity is shaped by the values and goals that revolve around this domain (Negru‐Subtirica et al. 2023). Schools are a crucial developmental and acculturative context (Schachner et al. 2018) in which adolescents not only acquire knowledge and develop their identity (Branje 2022; Verhoeven et al. 2019) but also experience a wide range of social and cultural diversity (Thijs and Verkuyten 2014). This is particularly true in modern school settings, where the presence of multiple generations of students with diverse ethnic and cultural backgrounds has made diversity more prominent than ever before (EUROSTAT 2025). These everyday encounters with difference offer adolescents the opportunity to develop social perspectives attuned to multicultural societies. In parallel, schools are crucial in transmitting civic values through both formal programs, such as civic and citizenship education, and informal socialization processes (Blasko et al. 2018; Luken Raz and Killen 2024). Through these experiences, adolescents at school can internalize principles of equality, justice, and respect for diversity, which can serve as a foundation for managing diversity and adapting to a multicultural society, thus shaping their level of intergroup solidarity.

How adolescents approach ethnic and cultural diversity and, in turn, the chances to develop intergroup solidarity can be influenced by their identity processes in the educational domain. In this regard, some evidence suggests that adolescents' in‐depth exploration of their educational identity is associated with lower prejudice toward other ethnic groups (Bobba et al. 2023). Therefore, actively reflecting on the importance of their academic choices serves as a protective factor for adolescents, reducing the development of negative intergroup attitudes and increasing the chances of showing greater solidarity. However, more research is needed to tackle how adolescents in different educational identity statuses could exhibit greater intergroup solidarity than others.

Furthermore, other factors, such as adolescents' ethnic background, might play a role in this association. Students from migrant families are more likely to be in the (searching) moratorium (Crocetti et al. 2011; Crocetti et al. 2008) or diffusion statuses (Schwartz et al. 2011) compared to their peers from the ethnic majority group, which, in turn, could impact their intergroup solidarity. In particular, research conducted in Europe has provided evidence of an “intensified identity work” approach (for a review, see Erentaitė et al. 2018), suggesting that ethnic minority youth are more engaged in identity work compared to their mainstream peers. In light of this, it is of utmost importance to understand how educational identity statuses are associated with adolescents' positive attitudes towards integration policies to better comprehend what can promote greater intergroup solidarity among adolescents with and without a migrant background.

### 2.4 The Current Study

Adolescence is crucial for embracing social perspectives and developing a more or less stable identity across different life domains, such as education. While abundant evidence links identity statuses to individual (e.g., well‐being) and interpersonal (e.g., family relationships) outcomes, research on social outcomes is limited. Consequently, it is still unclear whether educational identity statuses are associated with how adolescents perceive the diversity surrounding them. This study examined whether youth intergroup solidarity (i.e., positive attitudes toward migrant integration policies) differed over time based on their educational identity statuses (i.e., achievement, early closure, moratorium, searching moratorium, and diffusion). Furthermore, this study aims to understand if these differences are replicated among native Italian and migrant adolescents.

Specifically, it was hypothesized that adolescents in more stable identity statuses, such as achievement, would report higher levels of intergroup solidarity compared to peers in less stable statuses, consistent with prior evidence linking identity stable patterns to more positive psychosocial outcomes. It was also expected that adolescents with a migrant background might differ from their native Italian peers in terms of their representation in different identity statuses, which is in line with evidence of their “intensified identity work” approach. An exploratory approach was then adopted to investigate whether such differences also extend to the associations between their identity statuses and intergroup solidarity. Finally, although existing evidence on developmental trajectories of inclusive orientation is mixed, thus preventing clear predictions about possible positive or negative changes, or stability, over time, it was anticipated that differences in intergroup solidarity across identity statuses would remain consistent over time, reflecting the stable influence of identity development on adolescents' inclusive orientations.

## 3 Methods

### 3.1 Participants

Participants for this study were drawn from the ERC‐Consolidator longitudinal project IDENTITIES “Managing identities in diverse societies: A developmental intergroup perspective with adolescents”. A total of 1,547 adolescents (47.04% female, 52.96% male; *M*_age_ = 15.69 years, SD_age_ = 1.22, range: 13.69–20.04 years) participated in seven assessments (T1–T7) between 2022 and 2024. Adolescents were from two age groups: first‐year (44.81% female, 55.19% male; *M*_age_ = 14.66 years, SD_age_ = 0.53, range: 13.69–17.63 years) and third‐year (49.46% female, 50.54% male*; M*_age_ = 16.80 years, SD_age_ = 0.64, range: 15.74–20.04 years) students enroled in different secondary high schools. Specifically, the majority attended a lyceum (38.64%), which prepares students primarily for university studies. These youth were followed by those (37.80%) attending a technical school that offers specialized education aimed at higher education and direct entry into technical professions. Finally, 23.56% of students were enroled in vocational tracks focusing on practical skills and direct workforce entry after graduation. Schools were located in the Emilia‐Romagna region, in the North‐East of Italy, which is the region with the highest share of youth with a migrant background in the student population (Ministero della Pubblica Istruzione 2022). This was the main reason for choosing this area, given its relevance to the study of intergroup orientations.

Youth reported their parents' educational level, indicating that most parents had a medium (i.e., high school diploma; 47.52% fathers, 47.56% mothers), followed by those with a high (i.e., university degree; 23.98% fathers, 32.08% mothers) and a low (i.e., elementary or middle school degree; 28.50% fathers, 20.36% mothers) educational level. The parents' educational levels in the current sample are consistent with recent national (ISTAT 2024) and regional (Regione Emilia‐Romagna 2022) statistics for the population aged between 25 and 65.

Most adolescents (79.94%, 46.09% female, 53.91% male; *M*_age_ = 15.63 years, SD_age_ = 1.17, range: 13.69–20.04 years) were native Italians, while the remaining 20.06% (50.81% female, 49.19% male; *M*_age_ = 15.96 years, SD_age_ = 1.38, range: 13.87–19.40) had a migrant background. Migrant adolescents come from Eastern Europe (e.g., Albania; 34.17%), Asia (e.g., China; 27.84%), and Africa (e.g., Morocco; 25.32%), while the remaining come from America (e.g., Brazil; 7.60%), Western Europe (e.g., Germany; 3.80%), and Oceania (i.e., Australia; 1.27%). Most were second‐generation immigrants (73.76%), while the remaining 26.24% were first‐generation immigrants. The ethnic composition of the sample is consistent with recent national and regional statistics (Ministero della Pubblica Istruzione 2022).

Among the total sample, 24.37% of adolescents participated in all seven assessments, while 16.61%, 8.86%, 9.76%, 8.34%, 14.87%, and 17.19% participated in six, five, four, three, two, and one assessment, respectively. Thus, most participants (59.60% of the sample) completed more than half of the assessments (i.e., they participated in at least four out of seven assessments). To gain a clearer understanding of sample attrition, analyses were conducted to verify that attrition was not associated with specific variables. These analyses compared adolescents who participated in all seven assessments with those who completed six, five, four, three, two, or just one assessment. The results, presented in Supporting Information (see Tables S1a and S1b), indicated that these groups were largely comparable. The Little (1988) Missing Completely at Random (MCAR) test yielded a normed *χ* ^2^ (*χ* ^2^/df = 8149.477/6394) of 1.27, indicating that data were likely missing completely at random. Therefore, the total sample of 1547 participants was included in the analyses, and missing data were handled with the Full Information Maximum Likelihood (FIML) procedure in M*plus* 8.9 (Kelloway 2015; Muthén and Muthén 1988‐2017).

### 3.2 Procedures

The study was approved by the Ethics Committee of Alma Mater University of Bologna (Italy) as part of the ERC‐Consolidator project IDENTITIES “Managing identities in diverse societies: A developmental intergroup perspective with adolescents”. This longitudinal research involved adolescents from several high schools in the North‐East part of Italy. Schools were selected through a stratified (by track and level of urbanization) randomized method, and principals were approached to present the project. Upon their approval, the study was presented to students and their parents, who also received written and oral information about it. Active consent from parents and young adults was obtained before their children's participation, while underage adolescents provided their assent to participate in the project. Participation in the study was voluntary, and adolescents were informed they could withdraw their consent at any time. The seven data collections were conducted in January–February 2022 (T1), April–May 2022 (T2), September–October 2022 (T3), January–February 2023 (T4), April–May 2023 (T5), September–October 2023 (T6), January–February 2024 (T7).

### 3.3 Measures

Adolescents completed a questionnaire including socio‐demographics (e.g., age, sex, parents' educational level, and birth country) and measures of their intergroup solidarity (i.e., positive attitudes toward migrant integration policies) and their three educational identity processes (i.e., commitment, in‐depth exploration, and reconsideration of commitment). Information regarding the reliability of each measure is provided in the Supporting Information (see Table S2).

#### 3.3.1 Intergroup Solidarity

Adolescents' intergroup solidarity was assessed repeatedly across all seven waves using the Attitudes toward Migrant Integration Policies scale (AMIP; Maratia et al. 2024; Maratia and Crocetti 2024). The instrument consists of eight items based on the Migrant Integration Policy Index (MIPEX; Solano and Huddleston 2020). Participants received this prompt: “You will be presented with several policies for the integration of people with a migrant background. Please, rate how important it is that Italian national programs support policies to foster…” followed by one item for each policy area, as for example “…family reunion (e.g., accommodation, residence period)”. For each item, participants indicated their response on a 5‐point Likert scale (from 1 “Not at all important” to 5 “Absolutely important”).

#### 3.3.2 Educational Identity

Commitment, in‐depth exploration, and reconsideration of commitment in the educational domain were measured at T1 using the Utrecht‐Management of Identity Commitments Scale (U‐MICS, Crocetti et al. 2008; Italian validation by Crocetti et al. 2010). The instrument consists of 13 items scored on a 5‐point Likert scale, ranging from 1 (completely false) to 5 (completely true). Sample items include: “My education gives me certainty in life” (commitment; 5 items), “I think a lot about my education” (in‐depth exploration; 5 items), and “I often think it would be better to try to find a different education” (reconsideration of commitment; 3 items).

### 3.4 Strategy of Analyses

Descriptive analyses were conducted using IBM SPSS Version 28.0 for Windows. The remaining analyses were conducted in M*plus* 8.9 (Muthén and Muthén 1998‐2017), using the Maximum Likelihood Robust (MLR) estimator (Satorra and Bentler 2001).

Two preliminary steps were undertaken before conducting the main analyses. First, it was tested whether the measure of intergroup solidarity, the Attitudes toward Migrant Integration Policies scale (AMIP), showed longitudinal invariance. The full procedure is detailed in the Supplemental Materials. Second, data‐driven cluster analysis was conducted to identify adolescents in various educational identity statuses. This procedure was preferred over other methods, such as Latent Profile Analysis, to maintain consistency with the existing literature and allow for greater comparability, as cluster analysis has been the most widely employed approach for identifying identity statuses (e.g., Crocetti et al. 2008; Luyckx et al. 2008; Piotrowski et al. 2024). Specifically, the scores for the identity processes were standardized, and in the first step, a hierarchical cluster analysis was conducted. Cluster solutions with two, three, four, five, and six clusters were compared based on three criteria: theoretical meaningfulness of each cluster, parsimony, and explanatory power (i.e., the cluster solution had to explain approximately 50% of the variance in each identity dimension). In the second step, initial cluster centres obtained from the hierarchical cluster analysis were used as non‐random starting points in iterative *k*‐means clustering. This second step was applied to assign an identity status to each participant (Crocetti et al. 2014).

Regarding the main aim of this study, Latent Growth Curve (Duncan and Duncan 2009; Preacher 2018) analyses in M*plus* were conducted to examine mean‐level changes in intergroup solidarity among adolescents. Latent growth curve analyses allow the estimation of the intercept (i.e., the variable level at the beginning of the study) and the slope (i.e., the rate of change). Through the Satorra and Bentler's (2001) scaled difference chi‐square test statistic and changes in fit indices, three models (i.e., intercept–only model; linear model; free‐change model) were compared to determine which growth curve best captured observed changes in adolescents' intergroup solidarity (Preacher 2018). Multigroup Latent Growth Curve model and pairwise parameter comparisons using the Wald test were then conducted to examine differences in growth parameters based on adolescents' educational identity statuses.

As sensitivity analyses, first, Multigroup Latent Growth Curve analyses were conducted accounting for covariates such as sex, age group (i.e., first‐ vs. third‐year high school students), and socio‐economic status (i.e., parents' educational level). Second, to better understand the moderation role of ethnic background, the main analyses were performed separately for native Italian adolescents and those with a migrant background. Data, analysis codes, and outputs can be retrieved from the Open Science Framework (OSF) link https://doi.org/10.17605/OSF.IO/QDZ47.

## 4 Results

### 4.1 Preliminary Analyses

**Figure 1:**
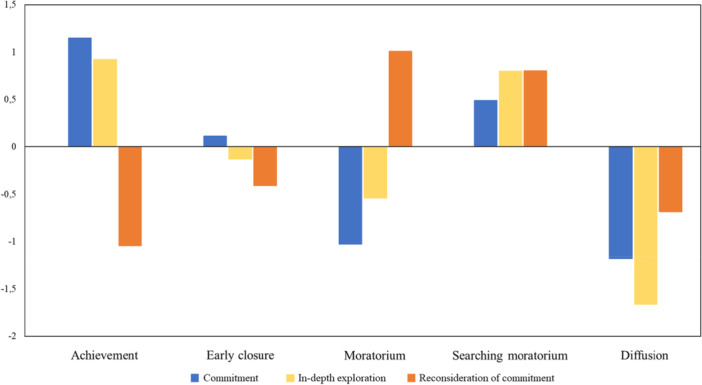
*Z*‐scores for commitment, in‐depth exploration, and reconsideration of commitment for the five educational identity statuses.

Means, standard deviation, and within‐time correlations for study variables are displayed in the Supporting Information (see Table S2). Regarding the longitudinal invariance of the intergroup solidarity measure, results indicated that partial scalar invariance was reached (see Table S3). Moving to the cluster analyses, the *z*‐scores of commitment, in‐depth exploration, and reconsideration of commitment for the final five clusters are shown in Figure 1. The first cluster (achievement, *n* = 183; 16.71% of the sample) comprised individuals who scored high on commitment and in‐depth exploration and low on reconsideration of commitment. The second cluster (early closure, *n* = 361; 32.97% of the sample) comprised individuals who scored moderately high on commitment, moderately low on in‐depth exploration, and low on reconsideration of commitment. The third cluster (moratorium, *n* = 233; 21.28% of the sample) consisted of adolescents who scored low on commitment and in‐depth exploration, but high on reconsideration of commitment. The fourth cluster (searching moratorium, *n* = 216; 19.73% of the sample) consisted of individuals scoring high on all identity processes. The fifth cluster (diffusion, *n* = 102; 9.31% of the sample) comprised individuals scoring low on all three dimensions. This five‐cluster solution explained 62%, 59%, and 62% of the variance in commitment, in‐depth exploration, and reconsideration of commitment, respectively.

### 4.2 Main Analyses

**Figure 2:**
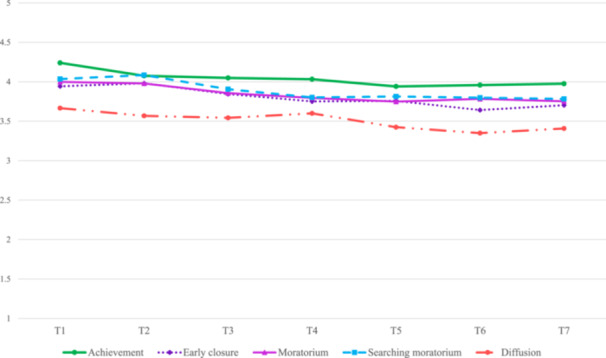
Estimated growth of intergroup solidarity of adolescents in different educational identity statuses.

The results of the Latent Growth Curve analyses indicated that the model that fitted the data significantly better was the free change model (see Table S4). Variances of attitudes toward migrant integration policies intercepts and slopes were statistically significant, indicating interindividual differences in levels and rates of change. Therefore, it was possible to conduct Multigroup Latent Growth Curve analyses to understand if adolescents' intergroup solidarity differed over time based on their educational identity. Estimated growth curves for the five clusters of educational identity are reported in Figure 2. Results of intercept comparisons (see Table 1) highlighted that adolescents in the achievement status showed more positive attitudes toward migrant integration policies compared to youth in the early closure (Wald test = 22.15, *p* < 0.001), moratorium (Wald test = 11.87, *p* < 0.001), searching moratorium (Wald test = 7.27, *p* < 0.01), and diffusion (Wald test = 49.62, *p* < 0.001) statuses. Adolescents in the early closure, moratorium, and searching moratorium statuses did not differ from each other and reported intermediate levels of intergroup solidarity. Adolescents in the diffusion status reported less favourable attitudes toward migrant integration compared to adolescents in the early closure (Wald test = 14.69, *p* < 0.001), moratorium (Wald test = 18.22, *p* < 0.001), and searching moratorium (Wald test = 19.54, *p* < 0.001) statuses. The slopes of the attitudes toward migrant integration policies did not differ among adolescents regardless of their identity statuses.

**Table 1:** Multigroup Latent Growth Curve analyses of intergroup solidarity.

Identity statuses	Growth factors	
	Intercept *M* (σ^2^)	Slope *M* (σ^2^)
Achievement status	$4.24_{a}^{***}$ (0.156**)	${-0.044}_{a}^{***}$ (0.004**)
Early closure status	$3.94_{b}^{***}$ (0.267***)	${-0.040}_{a}^{***}$ (0.003**)
Moratorium status	$4.00_{b}^{***}$ (0.291***)	${-0.041}_{a}^{***}$ (0.005**)
Searching moratorium status	$4.04_{b}^{***}$ (0.253**)	${-0.042}_{a}^{***}$ (0.004**)
Diffusion status	$3.67_{c}^{***}$ (0.212***)	${-0.043}_{a}^{*}$ (0.011*)

*Note: M* = mean; σ^2^ = variance.

Subscript letters within columns indicate significant coefficient differences, as emerged from the Wald test pairwise comparisons.

*p < 0.05; **p < 0.01; ***p < 0.001

### 4.3 Sensitivity Analyses

As sensitivity analyses, first, the Multigroup Latent Growth Curve Model was conducted by accounting for covariates such as sex, age group (i.e., first‐ vs. third‐year high school students), and socio‐economic status (i.e., parents' educational level). The results of this model confirmed the findings obtained in the model without covariate adjustments, highlighting the same initial differences in intergroup solidarity levels among adolescents based on their educational identity. Regarding the main effect of these covariates, concerning *sex*, results indicated that females (*b* = 0.056, *p* < 0.001) reported more positive attitudes toward migrant integration policies than males at the beginning of the study. However, no significant differences emerged in the rate of change in this form of intergroup solidarity over time based on adolescents' sex. Regarding the *age group* (i.e., first‐ vs. third‐year high school students), while results indicated no difference in the intercept of adolescents' intergroup solidarity, a difference emerged on its slope. In particular, older students (*b* = 0.005, *p* < 0.001) exhibited a more pronounced decline in their level of intergroup solidarity over time compared to younger students. Finally, regarding the *socio‐economic status* (i.e., parents' level of education), results indicated that students from higher socio‐economic (*b* = 0.082, *p* < 0.001) backgrounds reported higher levels of intergroup solidarity at the beginning of the study compared to youth from lower socio‐economic backgrounds. However, no significant differences emerged in the rate of change in intergroup solidarity over time based on youth socio‐economic status.

Second, analyses were conducted in the two subgroups of native Italian adolescents and those with a migrant background. Employing the Chi‐Square Test (*χ* ^2^(4) = 19.71, *p* = 0.001), significant differences in the distribution of adolescents with and without a migrant background along the five identity statuses were detected (see Table S6). Migrant adolescents were more likely to be in the searching moratorium status compared to their native Italian peers. Then, Multigroup Latent Growth Curve analyses were performed separately among adolescents with and without a migrant background. Importantly, the profile of the statuses in terms of showing more or less intergroup solidarity documented in the total sample was generally confirmed in both groups (for a detailed report, see Supporting Information, Table S7), indicating that adolescents in the achievement status showed the highest levels of intergroup solidarity, while the diffusion status exhibited the lowest.

## 5 Discussion

Integration policies are crucial for promoting intergroup solidarity and societal well‐being (Solano and Huddleston 2020). At the same time, their implementation appears intertwined with public opinion (for a review, see Baum and Potter 2008), making it important to understand how much individuals, especially adolescents, endorse these policies and which factors influence this form of intergroup solidarity. A large corpus of research shows that identity statuses are characterized by distinct profiles, especially in terms of personality, mental health, adjustment indicators, and quality of family and peer relationships (for a review, see Crocetti 2018). In contrast, less is known about how adolescents in different identity statuses differ in their social and intergroup relations. Given this knowledge gap, this study examined whether adolescents' intergroup solidarity differed over time based on the youth's various educational identity statuses. Overall, adolescents in the achievement status showed the highest levels of intergroup solidarity, while their peers in the diffusion status showed the lowest. These results were replicated among native Italian adolescents and those with a migrant background and highlighted the role of educational identity status in shaping adolescents' intergroup solidarity. Importantly, these findings should be interpreted in light of the broader socialization role of schools. Educational identity processes do not directly capture adolescents' endorsement of diversity‐related values. Rather, they reflect the extent to which adolescents engage with and invest in the educational domain. Given that schools represent key contexts where young people are exposed to civic values and social diversity, stronger engagement with the educational domain may foster greater reflection on and internalization of inclusive social orientations, which may in turn translate into greater intergroup solidarity.

### 5.1 Adolescents' Intergroup Solidarity and Educational Identity Statuses

The main differences based on adolescents' educational identity statuses concerned the initial levels of intergroup solidarity. Adolescents in the achievement status, who generally display the best profile in terms of personal characteristics (e.g., Morsunbul et al. 2016) and interpersonal relationships (e.g., Albert Sznitman et al. 2019), in the present study also showed better societal outcomes, exhibiting higher levels of intergroup solidarity than youth in other statuses. This result aligned with other evidence suggesting that adolescents with the achievement status show high social responsibility, demonstrating a strong desire to contribute to their communities (Crocetti et al. 2012). More stable identity patterns are indeed associated with greater civic engagement and prosocial values (e.g., Berzonsky and Kuk 2022), including pro‐diversity and pro‐equality values (Erentaitė et al. 2019) and increased openness to interreligious dialogue (Rydz and Romaneczko 2022). Thus, the enhanced clarity regarding themselves in the educational domain and their openness to experience may enable adolescents in the achievement status to navigate the surrounding world better, manifesting greater intergroup solidarity.

Youth in the early closure, moratorium, and searching moratorium statuses exhibited a similar profile. Thus, adolescents in the early closure, who typically share with their peers in the achievement status a similar profile in terms of psychological well‐being (Schwartz et al. 2011), differed from them in more complex matters like views and engagement towards the surrounding world (Crocetti et al. 2012), showing, in this case, slightly less intergroup solidarity. Adolescents in the moratorium status, often depicted negatively in terms of well‐being because of their high levels of anxiety and distress, especially compared to those in (early) closure (e.g., Albert Sznitman et al. 2019), when it comes to societal issues, they did not differ from the latter, indicating that, depending on the outcome, these adolescents can also show more adaptive patterns. Adolescents in the searching moratorium status, whose choices are often influenced by external pressure (Meeus et al. 2012), showed a favourable profile regarding intergroup solidarity, perhaps due to their high levels of in‐depth exploration, a process associated with less ethnic prejudice among youth (Bobba et al. 2023).

Adolescents in the diffusion status reported the lowest levels of intergroup solidarity compared to their peers in any other status. Thus, the less favourable outcomes these adolescents experience at the individual (e.g., Bogaerts et al. 2021; Hatano et al. 2016) and interpersonal (Albert Sznitman et al. 2019) levels of their lives were also confirmed from a broader perspective. These results might draw attention to evidence suggesting that these adolescents are less engaged socially, registering lower social responsibility (e.g., Crocetti et al. 2012). Similarly, adolescents who avoid identity issues may also be more vulnerable to extremist narratives (e.g., Isenhardt et al. 2021; Meeus 2015) and exhibit higher levels of intolerance (Ozer et al. 2023) and fearful globalization attitudes (Senejko and Łoś 2016). Overall, this result might reflect a disinterest that extends not only toward adolescents' identity commitments but also toward society and others, decreasing their sense of intergroup solidarity.

It should be emphasized that adolescents showed above‐average scores on the scale measuring attitudes toward integration policies, regardless of their identity status, thus providing a positive picture of their intergroup solidarity. This result appears consistent with other evidence from the Italian context, where adolescents typically report moderate‐to‐low negative attitudes toward individuals with a migrant background (e.g., Bobba et al. 2023). Thus, whenever differences between adolescents in various identity statuses are presented, they should be viewed in terms of greater or lesser intergroup solidarity. Furthermore, these five groups differed mainly in the initial levels of intergroup solidarity but not in the rate of change, which showed a similar negative trend across all adolescents. This suggests that broader developmental and contextual factors may contribute to the overall decline in inclusive attitudes over time. For instance, as adolescents grow older and increasingly focus on their future career pathways, perceptions of migrants as potential competitors for socio‐economic opportunities may become more salient (Mitchell 2019), thus hindering their level of intergroup solidarity. Moreover, the heightened political awareness that youth develop during adolescence (Rekker et al. 2015) may lead to more sharply defined stances toward integration policies, whether positively or negatively. Additionally, shifts in national politics, which notably occurred also in Italy during the study period, may have further influenced these attitudes, as highlighted by recent evidence suggesting that even slight changes at the national level can shape the political orientations of youth undergoing political socialization (Jeannet and Dražanová 2024).

However, despite this general decline, the overall pattern of differences between identity statuses remained clearly visible, with achievement and diffusion consistently representing the two poles of the spectrum and the intermediate statuses positioned between them. This stability across time suggests that educational identity continues to shape adolescents' social orientations, even as broader developmental and contextual changes tend to reduce intergroup solidarity during adolescence. Furthermore, although this form of intergroup solidarity decreased over time, mean scores remained above average, providing a similar picture compared to the beginning of the study, with the differences between the various statuses still visible.

### 5.2 What About the Role of Demographic Drivers on Youth's Intergroup Solidarity?

Demographic characteristics were found to be related to intergroup solidarity. Sex differences were detected, with females reporting more positive attitudes toward migrant integration policies than males. This aligns with evidence suggesting that girls tend to be more inclusive, possibly due to gender‐specific socialization encouraging other‐oriented attitudes and greater empathic concerns (Dozo 2015; Van der Graaff et al. 2014).

As for age, while no difference was found in the initial level of adolescents' intergroup solidarity, a difference in the slope indicated that older students exhibited a more pronounced decline in their level of intergroup solidarity over time than younger students. On the one hand, the lack of differences in the intercepts may reflect the competing effects of cognitive maturation (Bayram Özdemir et al. 2021) and decreased social trust (Flanagan and Stout 2010), which contribute to a general stability of intergroup solidarity across age groups (for a meta‐analysis, see Crocetti et al. 2021). On the other hand, the differences in the slopes may be explained by the more salient transition to the job market experienced by older adolescents, which could heighten perceptions of outgroups as potential threats to their future socio‐economic opportunities (Mitchell 2019).

Finally, family socio‐economic status seems to positively affect adolescents' inclusive orientations, with youth whose parents have higher education levels showing greater intergroup solidarity. These results align with previous findings linking higher socio‐economic status to stronger pro‐immigrant attitudes (Dražanová et al. 2023). Overall, these demographic factors provide essential context for understanding the variability and development of adolescents' intergroup solidarity.

#### 5.2.1 Zooming in on Italian Native Adolescents and Their Peers With a Migrant Background

This study also explored whether the results were replicated among native Italian adolescents and those with a migrant background. Regarding potential differences in the distribution of various identity statuses, migrant adolescents were more likely to be in the searching moratorium status than their native Italian peers. This result aligns with other evidence that finds the same differences between ethnic majority and minority families (e.g., Crocetti et al. 2011) and can be explained by the more intensive identity crisis these adolescents experience across different identity domains (e.g., ethnic and educational), compared to youth from the ethnic majority group (for a review, see Erentaitė et al. 2018).

While differences in the distribution across the various identity statuses were found, overall, native Italian adolescents and those with a migrant background did not differ in their level of intergroup solidarity based on their educational identity status. This result is consistent with previous findings on the topic, which similarly reported no significant differences in positive attitudes toward integration policies between these two groups (Maratia et al. 2024). Indeed, for both groups, the achievement and diffusion statuses represented the two poles of the spectrum, with the achievement status associated with more favourable attitudes toward migrant integration policies and the diffusion status linked to less intergroup solidarity. In addition, the distinction between these two extreme statuses (i.e., achievement and diffusion) and the intermediate ones (i.e., early closure, moratorium, and searching moratorium), as well as the decline in intergroup solidarity over time, was less pronounced among adolescents with a migrant background. On the one hand, these subtle differences may stem from the smaller sample size of migrant adolescents, compared to native Italians, which may limit the ability to detect clearer patterns. On the other hand, the greater personal relevance of integration policies for these youths might reduce the differences in intergroup solidarity across various identity statuses, particularly among those in intermediate groups. Overall, these results thus demonstrated that youth intergroup solidarity is associated with their educational identity, regardless of their ethnic background.

### 5.3 Practical Implications: From Individual to Societal Well‐Being

The current study has important practical implications, especially in the school context, where adolescents develop cognitive skills and pursue academic goals (for a review, see York et al. 2019), while shaping orientations toward the surrounding world (Schwarzenthal et al. 2018). Schools, therefore, serve as the foundation where the citizens of tomorrow are shaped, and their future adult perspectives are formed (Rekker et al. 2015). This study has shown that the development of adolescents' identity within this specific domain can have far‐reaching effects, fostering more intergroup solidarity among adolescents. As such, the research underscored the importance of interventions promoting educational or vocational identity development (for a meta‐analysis, see Crocetti et al. 2025). These interventions would not only support academic achievement and more stable career paths for youth (e.g., Sugimura et al. 2023) but also encourage greater intergroup solidarity among adolescents, both with and without a migrant background. At the same time, such interventions could generate a virtuous cycle effect (Levantini et al. 2026), indirectly facilitating the implementation of integration policies and their benefits in terms of greater cohesion (e.g., Green et al. 2020; Schlueter et al. 2020) and well‐being (e.g., Sand and Gruber 2018; Tatarko et al. 2021) at the broader societal level.

### 5.4 Strengths, Limitations, and Suggestions for Future Research

This study, which contributed to disentangling the development of intergroup solidarity (i.e., positive attitudes toward migrant integration policies) among adolescents within different educational identity statuses, should be considered in light of its strengths and limitations, which suggest future research directions. A key strength of this study was its focus on the school environment, a crucial setting for adolescents' identity formation (Negru‐Subtirica et al. 2023). However, identity unfolds across multiple domains (Dimitrova et al. 2018), which this study did not consider. Thus, it could be important to explore how other identity domains, such as the interpersonal one, given the crucial role of peer relationships for adolescents (Ragelienė 2016), might also influence the development of intergroup solidarity among youth. Moreover, beyond individual characteristics, other factors related to the school context itself (Albarello et al. 2023), such as the quality of peer interactions (Bohman and Kudrnáč 2023), the frequency and nature of intergroup contact (Karataş et al. 2023), and the educational content promoted (Blasko et al. 2018), may play a key role in shaping the development of intergroup solidarity. Future studies should therefore consider these additional dimensions of both individuals' identity and the school context to gain a more comprehensive understanding of the processes that foster or hinder adolescents' positive social orientations.

Additionally, since the study focused on the development of intergroup solidarity, it was conducted in the Italian region (i.e., Emilia‐Romagna) with the highest ethnic diversity in the school population (Ministero della Pubblica Istruzione 2022). This setting allowed for a nuanced exploration of individuals' intergroup relations and solidarity (Spitzerová et al. 2025; Verkuyten 2022). However, this choice introduced a limitation, as the findings may not be fully generalizable to less diverse school environments. Consequently, more research in other contexts with different degrees of ethnic and cultural diversity is needed.

Furthermore, the longitudinal design represented a major strength, providing insights into how intergroup solidarity evolved and varied according to youth educational identity statuses. However, other factors that could account for differences in the rate of change of youth solidarity were not considered (e.g., increased threat; Mitchell 2019). This limited a more comprehensive understanding of other processes involved, especially considering that adolescents showed decreased intergroup solidarity over time, regardless of their educational identity status. Thus, future studies could focus on identifying factors beyond educational identity that may influence the development of intergroup solidarity during adolescence.

## 6 Conclusions

While the link between identity statuses and adolescents' individual and interpersonal outcomes has been consistently investigated, less is known about whether and how identity development affects their level of intergroup solidarity. This study highlighted the association between educational identity status and adolescents' attitudes toward migrant integration policies, indicating that adolescents in the achievement status showed the highest level of intergroup solidarity, while those in the diffusion status reported the lowest. These results were replicated among adolescents with and without a migrant background. This study highlighted that youth intergroup solidarity is associated with adolescents' educational identity. Thus, knowing themselves in this domain and deeply exploring the importance of what they study can help youth navigate the diversity of the surrounding world and foster higher intergroup solidarity.

## Author Contributions

**Fabio Maratia:** conceptualization, data curation, formal analysis, investigation, methodology, writing – original draft preparation. **Elisabetta Crocetti:** conceptualization, funding acquisition, methodology, resources, supervision, writing – original draft preparation.

## Ethics Statement

All procedures performed in this study involving human participants were in accordance with the ethical standards of the Ethics Committee of the Alma Mater Studiorum University of Bologna (Italy) and with the 1964 Helsinki declaration and its later amendments or comparable ethical standards.

## Consent

Active consent was obtained from parents for underage adolescents, while older youth provided their own active consent to participate in the project. Underage youth also provided their assent to take part in the study.

## Conflicts of Interest

The authors declare no conflicts of interest.

## Supporting information


Supporting File
