# Supplementary material for: If I Know Myself, I Can Welcome You: Identity Roots of Intergroup Solidarity
Source: J Adolesc. 2026 Apr 12;98(5):1465–78. doi: 10.1002/jad.70154 (PMC13338656; doi:10.1002/jad.70154)
Supplement: Supplementary file 1 — Supporting File [file JAD-98-1465-s001.docx]

**Supplemental Materials for**

**If I Know Myself, I Can Welcome You: Identity Roots of Intergroup Solidarity**

[**Sample Attrition Analyses** 3](#_Toc205461734)

[**Table S1a.** 4](#_Toc205461735)

[**Table S1b.** 5](#_Toc205461736)

[**Table S2** 6](#_Toc205461737)

[Cronbach's Alphas, means, standard deviations, and correlations among study variables. 6](#_Toc205461738)

[**Longitudinal Measurement Invariance of Intergroup Solidarity** 8](#_Toc205461739)

[**Table S3** 9](#_Toc205461740)

[Longitudinal invariance of the measure of intergroup solidarity 9](#_Toc205461741)

[**Table S4.** 10](#_Toc205461742)

[Latent Growth Curve analyses of adolescents' intergroup solidarity 10](#_Toc205461743)

[**Table S5.** 11](#_Toc205461744)

[Observed means and standard deviations of participants' intergroup solidarity based on their educational identity statuses. 11](#_Toc205461745)

[**Sensitivity analyses** 12](#_Toc205461746)

[***Moderation by Ethnic Background*** 12](#_Toc205461747)

[**Table S6.** 14](#_Toc205461748)

[Native Italian and migrant adolescents' distribution in each educational identity status 14](#_Toc205461749)

[**Table S7.** 15](#_Toc205461750)

[Latent Growth Curve analyses of native Italian and migrant adolescents' intergroup solidarity 15](#_Toc205461751)

[**Table S8.** 16](#_Toc205461752)

[Multigroup Latent Growth Curve analyses of intergroup solidarity 16](#_Toc205461753)

[**Figure S1.** 17](#_Toc205461754)

[Estimated growth of intergroup solidarity of native Italian adolescents in different educational identity statuses 17](#_Toc205461755)

[**Figure S2.** 18](#_Toc205461756)

[Estimated growth of intergroup solidarity of migrant adolescents in different educational identity statuses 18](#_Toc205461757)

# **Sample Attrition Analyses**

To gather a better understanding of the sample attrition, additional analyses were completed to ensure that the attrition was not related to specific variables by confronting adolescents who participated in all seven assessments with those who attended only six, five, four, three, two, or one assessment.

First, the effect of demographic variables was examined. No significant differences emerged for adolescents' sex and age. Regarding ethnic background, youth with a migrant background were overrepresented among those who completed the questionnaire in one or two waves, and underrepresented among those who completed all seven waves. Conversely, native Italian adolescents were overrepresented among participants who completed all seven waves. As for socioeconomic status, few differences emerged. In particular, adolescents whose fathers had a high level of education (i.e., university degree) were overrepresented among those who participated in all seven waves. Conversely, adolescents whose mothers had a medium educational level (i.e., high school diploma) were underrepresented among those who participated in only one wave, whereas those whose mothers had a high educational level were overrepresented among participants who completed all seven waves. Detailed results are presented in Table S1a.

Regarding the main study variables, results were broadly comparable **except for a few differences.** For educational identity processes at T1, adolescents who participated in only one wave reported lower mean levels of commitment and in-depth exploration than those who participated in six and seven waves, respectively. Regarding intergroup solidarity across the seven waves, adolescents who completed all seven waves reported higher levels of inclusive attitudes than those who participated in four, three, two, and one waves. Similarly, those who participated in six waves scored higher than those who completed four and two waves. Detailed results are available in Table S1b. The explained variance was very small (around 1-2%). **Overall, these findings suggest that the study's results are unlikely to be undermined by selective attrition.**

# **Table S1a.**

Sample attrition analyses for demographic variables

|  | **Participated in** | | | | | | | |  | |  | |  |  |
| --- | --- | --- | --- | --- | --- | --- | --- | --- | --- | --- | --- | --- | --- | --- |
|  | **7 waves**  **(*n* = 377)** | **6 waves**  **(*n* = 257)** | **5 waves**  **(*n* = 137)** | **4 waves**  **(*n* = 151)** | **3 waves**  **(*n* = 129)** | **2 waves**  **(*n* = 230)** | **1 wave**  **(*n* = 266)** |  | |  | |  | |  |
|  | ***n* (%)** | ***n* (%)** | ***n* (%)** | ***n* (%)** | ***n* (%)** | ***n* (%)** | ***n* (%)** | ***χ*^2^** | | **df** | | ***p*** | | **Cramers’V** |
| **Sex** |  |  |  |  |  |  |  | **20.368** | | **6** | | **<.01** | | **.115** |
| *Boys* | 188 (50) | 118 (46.09) | 66 (48.18) | 89 (59.33) | 82 (64.57) | 133 (59.11) | 135 (51.53) |  | |  | |  | |  |
| *Girls* | 188(50) | 138 (53.91) | 71 (51.82) | 61 (40.67) | 45 (35.43) | 92 (40.89) | 127 (48.47) |  | |  | |  | |  |
|  |  |  |  |  |  |  |  |  | |  | |  | |  |
| **Background** |  |  |  |  |  |  |  | **55.497** | | **6** | | **<.001** | | **.197** |
| *Native Italians* | **332(+)** (86.06) | 205 (83) | 101 (78.91) | 94 (75.81) | 86 (75.44) | 138 (66.03) | 164 (68.91) |  | |  | |  | |  |
| *Migrants* | **45(-)** (11.94) | 42 (17) | 27 (21.09) | 30 (24.19) | 28 (24.56) | **71(+)** (33.97) | **74(+)** (31.09) |  | |  | |  | |  |
|  |  |  |  |  |  |  |  |  | |  | |  | |  |
| **Age** |  |  |  |  |  |  |  | 8.995 | | 6 | | >.05 | | .076 |
| *First cohort* | 189 (50.27) | 133 (51.95) | 63 (45.99) | 93 (62) | 68 (52.71) | 117 (51.09) | 143 (54.17) |  | |  | |  | |  |
| *Second cohort* | 187 (49.73) | 123 (48.05) | 74 (54.01) | 57 (38) | 61 (47.29) | 112 (48.91) | 121 (45.83) |  | |  | |  | |  |
|  |  |  |  |  |  |  |  |  | |  | |  | |  |
| **Fathers’ education** |  |  |  |  |  |  |  | **31.345** | | **12** | | **<.01** | | **.154** |
| *Low* | 91 (55.32) | 60 (24.79) | 45 (37.50) | 29 (27.36) | 37 (34.91) | 47 (24.61) | 69 (35.94) |  | |  | |  | |  |
| *Medium* | 167 (34.57) | 121 (50) | 56 (46.67) | 57 (53.77) | 49 (46.23) | 104 (54.45) | 77 (40.10) |  | |  | |  | |  |
| *High* | **112(+)** (10.11) | 61 (25.21) | 19 (15.83) | 20 (18.87) | 20 (18.87) | 40 (20.94) | 46 (23.96) |  | |  | |  | |  |
|  |  |  |  |  |  |  |  |  | |  | |  | |  |
| **Mothers’ education** |  |  |  |  |  |  |  | **60.026** | | **12** | | **<.001** | | **.213** |
| *Low* | **48(-)** (13.01) | **29(-)** (12.03) | 24 (19.83) | 22 (20.56) | 30 (28.30) | 51 (26.42) | **67(+)** (34.90) |  | |  | |  | |  |
| *Medium* | 181 (49.05) | 133 (55.19) | 60 (49.59) | 50 (46.73) | 47 (44.34) | 90 (46.63) | **71(-)** (36.98) |  | |  | |  | |  |
| *High* | **140(+)** (37.94) | 79 (32.78) | 37 (30.58) | 35 (32.71) | 29 (27.36) | 52 (26.94) | 54 (28.13) |  | |  | |  | |  |

*Note.* Parents’ educational levels: Low = Elementary or middle school degree; Medium = High school diploma; High: University degree. Observed values indicated in bold are significantly different from expected values: [+] indicates that the observed value is higher than the expected value; [-] indicates that the observed value is lower than the expected value.

# **Table S1b.**

Sample attrition analyses for main variables

|  | **Participated in** | | | | | | | | | | | | | |  |  |  |
| --- | --- | --- | --- | --- | --- | --- | --- | --- | --- | --- | --- | --- | --- | --- | --- | --- | --- |
|  | **7 waves**  **(*n* = 377)** | | **6 waves**  **(*n* = 257)** | | **5 waves**  **(*n* = 137)** | | **4 waves**  **(*n* = 151)** | | **3 waves**  **(*n* = 129)** | | **2 waves**  **(*n* = 230)** | | **1 wave**  **(*n* = 266)** | |  |  |  |
|  | ***M*** | ***SD*** | ***M*** | ***SD*** | ***M*** | ***SD*** | ***M*** | ***SD*** | ***M*** | ***SD*** | ***M*** | ***SD*** | ***M*** | ***SD*** | ***F*** | ***p*** | **η^2^** |
| Commitment | 3.21_ab_ | 0.77 | 3.25_a_ | 0.78 | 3.15_ab_ | 0.97 | 3.10_ab_ | 0.78 | 3.01_ab_ | 0.99 | 3.08_ab_ | 0.81 | 2.90_b_ | 0.89 | 3 | <.01 | .016 |
| In-depth exploration | 3.20_a_ | 0.66 | 3.14_ab_ | 0.61 | 3.21_ab_ | 0.77 | 3.08_ab_ | 0.71 | 2.98_ab_ | 0.78 | 3.04_ab_ | 0.70 | 2.97_b_ | 0.68 | 3.042 | <.01 | .016 |
| Reconsideration of commitment | 2.90 | 0.83 | 2.88 | 0.84 | 2.87 | 0.99 | 3.10 | 0.95 | 2.97 | 0.91 | 3.12 | 0.84 | 3.12 | 0.98 | 2.509 | <.05 | .014 |
|  |  |  |  |  |  |  |  |  |  |  |  |  |  |  |  |  |  |
| Intergroup solidarity | 3.94_a_ | 0.55 | 3.91_ac_ | 0.53 | 3.83_abc_ | 0.65 | 3.69_b_ | 0.57 | 3.72_bc_ | 0.58 | 3.74_b_ | 0.60 | 3.76_bc_ | 0.80 | 6.261 | <.001 | .025 |

*Note.* Means for intergroup solidarity (i.e., attitudes toward migrant integration policies) were aggregated across the seven waves. Different subscript letters indicate significant differences between means (*p* < .05) as indicated by Tukey's HSD post hoc tests.

# **Table S2**

# Cronbach's Alphas, means, standard deviations, and correlations among study variables.

|  | α | *M* | *SD* | 1. | 2. | 3. | 4. | 5. | 6. | 7. | 8. | 9. | 10. |
| --- | --- | --- | --- | --- | --- | --- | --- | --- | --- | --- | --- | --- | --- |
| 1. Background |  |  |  | - |  |  |  |  |  |  |  |  |  |
| 2. Commitment T1 | .887 | 3.14 | 0.83 | .01 | - |  |  |  |  |  |  |  |  |
| 3. In-depth exploration T1 | .739 | 3.13 | 0.68 | -.00 | .57^***^ | - |  |  |  |  |  |  |  |
| 4. Reconsideration of commitment T1 | .773 | 2.96 | 0.88 | .13^***^ | -.30^***^ | -.23 | - |  |  |  |  |  |  |
| 5. Intergroup solidarity T1 | .911 | 4.00 | 0.73 | .02 | .13^***^ | .22^***^ | -.14 | - |  |  |  |  |  |
| 6. Intergroup solidarity T2 | .869 | 3.98 | 0.65 | .05 | .13^***^ | .30^***^ | -.01 | .54^***^ | - |  |  |  |  |
| 7. Intergroup solidarity T3 | .898 | 3.91 | 0.68 | .03 | .17^***^ | .20^***^ | .02 | .48^***^ | .54^***^ | - |  |  |  |
| 8. Intergroup solidarity T4 | .912 | 3.81 | 0.71 | .02 | .13^***^ | .18^***^ | -.05 | .55^***^ | .54^***^ | .66^***^ | - |  |  |
| 9. Intergroup solidarity T5 | .901 | 3.79 | 0.71 | .05 | .13^***^ | .19^***^ | -.05 | .50^***^ | .55^***^ | .63^***^ | .65^***^ | - |  |
| 10. Intergroup solidarity T6 | .920 | 3.75 | 0.73 | -.05 | .10^***^ | .18^***^ | -.02 | .50^***^ | .50^***^ | .61^***^ | .61^***^ | .64^***^ | - |
| 11. Intergroup solidarity T7 | .915 | 3.78 | 0.73 | .12 | .12^***^ | .19^***^ | -.03 | .52^***^ | .52^***^ | .61^***^ | .63^***^ | .68^***^ | .73^***^ |

*Note*. α=Cronbach's Alphas; *M*=Means; *SD*=Standard Deviations; Background: Adolescents' ethnic background; Background: 0=Italian, 1=Migrant.

Intergroup Solidarity=Positive attitudes toward migrant integration policies; T=Time.

^***^ *p* < .001

# **Longitudinal Measurement Invariance of Intergroup Solidarity**

As a preliminary step, it was tested whether the measure of intergroup solidarity, the Attitudes toward Migrant Integration Policies scale (AMIP), showed longitudinal invariance. For longitudinal configural, metric, and scalar levels were tested. To this end, the configural models function as baseline models and should therefore display a good fit, evaluated based on the following criteria: the Comparative Fit Index (CFI) with values higher than .90 and .95 indicative of an acceptable and very good fit; the Root Mean Square Error of Approximation (RMSEA) and the Standardized Root Mean Residual (SRMR) with values below .08 and .05 indicative of an acceptable and very good fit (Byrne, 2012); and the RMSEA's 90% confidence interval's upper bound lower than .10 indicating an acceptable fit of the model (Chen et al., 2008). To establish scalar invariance (i.e., constraining factor loadings and intercepts to be equal across time and/or groups), changes in fit indices from the configural to the metric and scalar model were evaluated (e.g., Cheung & Rensvold, 2002). Specifically, a significant Δχ_SB_^2^ (Satorra & Bentler, 2001), and ΔCFI ≥ -.010 supplemented by ΔRMSEA ≥ .015 (Chen, 2007) indicates non-invariance. Results are displayed in Table S3. As can be inferred, partial scalar invariance was reached.

# **Table S3**

# Longitudinal invariance of the measure of intergroup solidarity

| Models |  | | | | |  | Model comparisons | | | | | |
| --- | --- | --- | --- | --- | --- | --- | --- | --- | --- | --- | --- | --- |
|  | χ^2^ | df | CFI | SRMR | RMSEA [90% CI] |  | Models | Δχ_SB_^2^ | ΔCFI | ΔRMSEA | |  |
| Longitudinal Invariance | | | | | | | | | | | | |
| Configural (M1) | 3060.069 | 1295 | .927 | .047 | .030 [.029, .032] |  |  |  |  | |  | |
| Metric (M2) | 3163.236 | 1337 | .924 | .052 | .030 [.029, .032] |  | M2-M1 | 104.148 (42)^***^ | -.003 | | .000 | |
| Full scalar (M3_a_) | 3547.613 | 1385 | .910 | .064 | .033 [.031, .034] |  | M3_a_-M1 | 449.098 (48)^***^ | -.014 | | .003 | |
| Partial scalar (M3_b_) | 3416.044 | 1361 | .915 | .062 | .032 [.031, .033] |  | M3_b_-M1 | 313.958 (24)^***^ | -.009 | | .002 | |

*Note*. M = model; χ^2^ = chi-square; df = degree of freedom; CFI = Comparative Fit Index; SRMR = Standardized Root Mean Square Residual;

RMSEA = Root Mean Square Error of Approximation; CI = confidence interval; Δ = change in the parameter. The row highlighted in gray indicates the selected model following the comparison. Following standard procedures for partial scalar invariance testing, equality constraints on two item intercepts were released.

^***^ *p* < .001

# **Table S4.**

# Latent Growth Curve analyses of adolescents' intergroup solidarity

|  | Growth Factors | |  | Model fit | | | | |  | Model comparisons | |  |  |
| --- | --- | --- | --- | --- | --- | --- | --- | --- | --- | --- | --- | --- | --- |
|  | Intercept *M* (σ^2^) | Slope *M* (σ^2^) |  | χ^2^ | *df* | CFI | TLI | RMSEA [90% CI] |  | Models | Δχ_SB_^2^ (Δ*df*) | ΔCFI | ΔRMSEA |
|  | |  |  |  |  |  |  |  |  |  |  |  |  |
| M1: Intercept–only model | 3.829^***^ (.285^***^) |  |  | 258.331 | 26 | .829 | .862 | .078 [.069, .087] |  |  |  |  |  |
| M2: Linear model | 3.967^***^ (.258^***^) | $-$.051^***^ (.005^***^) |  | 50.085 | 23 | .980 | .982 | .028 [.018, .039] |  | M2–M1 | 181.942 (3)^***^ | -0.151 | 0.050 |
| M3: Free–change model | 3.963^***^ (.263^***^) | $-$.044^***^ (.004^***^) |  | 24.418 | 18 | .995 | .994 | .016 [.000, .030] |  | M3–M1 | 25.135 (5)^***^ | -0.015 | 0.012 |

*Note*. *M*=Mean; σ^2^= Variance; χ^2^= Chi-square; *df*= Degrees of freedom; CFI= Comparative Fit Index; TLI= Tucker–Lewis Index; RMSEA= Root Mean Square Error of Approximation and 90% confidence interval; Δ= change in parameter. The row highlighted in gray indicates the selected model following the comparison.

^***^ *p* < .001

# **Table S5.**

# Observed means and standard deviations of participants' intergroup solidarity based on their educational identity statuses.

|  | T1 | |  | T2 | |  | T2 | |  | T4 | |  | T5 | |  | T6 | |  | T7 | |
| --- | --- | --- | --- | --- | --- | --- | --- | --- | --- | --- | --- | --- | --- | --- | --- | --- | --- | --- | --- | --- |
|  | *M* | *SD* |  | *M* | *SD* |  | *M* | *SD* |  | *M* | *SD* |  | *M* | *SD* |  | *M* | *SD* |  | *M* | *SD* |
| Achievement | 4.25 | 0.63 |  | 4.10 | 0.70 |  | 4.10 | 0.69 |  | 4.06 | 0.69 |  | 4.04 | 0.67 |  | 4.01 | 0.72 |  | 4.05 | 0.72 |
| Early Closure | 3.94 | 0.68 |  | 4.00 | 0.62 |  | 3.90 | 0.65 |  | 3.81 | 0.69 |  | 3.80 | 0.67 |  | 3.72 | 0.71 |  | 3.80 | 0.70 |
| Moratorium | 4.00 | 0.75 |  | 3.96 | 0.63 |  | 3.91 | 0.72 |  | 3.80 | 0.77 |  | 3.77 | 0.75 |  | 3.77 | 0.78 |  | 3.73 | 0.76 |
| Searching Moratorium | 4.08 | 0.73 |  | 4.09 | 0.61 |  | 3.99 | 0.61 |  | 3.87 | 0.67 |  | 3.87 | 0.69 |  | 3.81 | 0.63 |  | 3.90 | 0.71 |
| Diffusion | 3.60 | 0.84 |  | 3.63 | 0.69 |  | 3.58 | 0.63 |  | 3.59 | 0.75 |  | 3.55 | 0.93 |  | 3.56 | 0.88 |  | 3.60 | 0.80 |

*Note*. T=Time; *M*=Means; *SD*=Standard deviation.

# **Sensitivity analyses**

## ***Moderation by Ethnic Background***

To better understand the moderation role of ethnic background, first, the cluster distribution of native Italian and migrant adolescents was assessed (see Table S6). Second, Multigroup Latent Growth Curve analyses were performed separately for native Italian adolescents and those with a migrant background. The results of the Latent Growth Curve analyses indicated that the model fitting the data significantly better for native Italian adolescents and those with a migrant background was the free change model (see Table S7). However, when conducting multigroup analyses for migrant adolescents, the free-change and linear models failed to converge. Therefore, the more parsimonious intercept-only model was adopted for this sub-sample. Estimated growth curves for the five clusters of educational identity in the two sub-samples are reported in Figures S1 and S2.

Results of intercept comparisons (see Table S8), highlighted that *native Italian adolescents* in the achievement status reported the highest level of intergroup solidarity, showing more positive attitudes toward migrant integration policies compared to youth in the early closure (Wald test=23.95, *p* < .001), moratorium (Wald test=14.33, *p* < .001), searching moratorium (Wald test=7.12, *p* < .01), and diffusion (Wald test=50.43, *p* < .001) statuses. Furthermore, native Italian adolescents in the early closure, moratorium, and searching moratorium statuses did not differ from each other and reported intermediate levels of intergroup solidarity. In addition, native Italian adolescents in the diffusion status of their educational identity reported the lowest level of intergroup solidarity, showing less favorable attitudes toward migrant integration policies compared to adolescents in the early closure (Wald test=13.88, *p* < .001), moratorium (Wald test=13.73, *p* < .001), and searching moratorium (Wald test=16.85, *p* < .001) statuses.

Regarding *adolescents with a* *migrant background*, although youth in the achievement status differed from those in the diffusion status (Wald test=5.05, *p* < .05), showing a higher level of intergroup solidarity than the latter, they did not differ from adolescents in the early closure, moratorium, and searching moratorium statuses. At the same time, adolescents in the early closure, moratorium, and searching moratorium statuses did not differ from each other. Additionally, adolescents in the diffusion status differed from those in the moratorium status (Wald test=4.28, *p* < .05), showing lower levels of intergroup solidarity than the latter, but they did not differ from youth in the early closure and searching moratorium statuses. Finally, the slopes of the attitudes toward migrant integration policies did not differ among native Italian adolescents and those with a migrant background, regardless of their identity statuses.

# **Table S6.**

# Native Italian and migrant adolescents' distribution in each educational identity status

|  | Native Italian Adolescents | |  | | Migrant Adolescents | |
| --- | --- | --- | --- | --- | --- | --- |
| Statuses | *n* (%) |  | | *n* (%) | |  |
| Achievement | 158 (17.48) |  | | 25 (13.09) | |  |
| Early Closure | 312 (34.51) |  | | 49 (25.65) | |  |
| Moratorium | 189 (20.91) |  | | 44 (23.04) | |  |
| Searching Moratorium | 158 (17.48) |  | | **58 (30.37) (+)** | |  |
| Diffusion | 87 (9.62) |  | | 15 (7.85) | |  |

*Note.* Observed values indicated in bold are significantly different from expected values: (+) indicates that the observed value is higher than the expected value.

# **Table S7.**

# Latent Growth Curve analyses of native Italian and migrant adolescents' intergroup solidarity

|  | Growth Factors | |  | Model fit | | | | |  | Model comparisons | |  |  |
| --- | --- | --- | --- | --- | --- | --- | --- | --- | --- | --- | --- | --- | --- |
|  | Intercept *M* (σ^2^) | Slope *M* (σ^2^) |  | χ^2^ | *df* | CFI | TLI | RMSEA [90% CI] |  | Models | Δχ_SB_^2^ (Δ*df*) | ΔCFI | ΔRMSEA |
| ***Native Italian Adolescents*** | |  |  |  |  |  |  |  |  |  |  |  |  |
| M1: Intercept–only model | 3.815^***^ (.285^***^) |  |  | 224.920 | 26 | .830 | .863 | .080 [.071, .090] |  |  |  |  |  |
| M2: Linear model | 3.957^***^ (.251^***^) | $-$.051^***^ (.005^***^) |  | 39.283 | 23 | .986 | .987 | .024 [.010, .037] |  | M2–M1 | 173.433 (3)^***^ | -0.156 | 0.056 |
| M3: Free–change model | 3.960^***^ (.255^***^) | $-$.045^***^ (.004^***^) |  | 17.504 | 18 | 1.00 | 1.00 | .000 [.000, .025] |  | M3–M1 | 21.933 (5)^***^ | -0.014 | 0.024 |
| ***Adolescents with a migrant background*** | | |  |  |  |  |  |  |  |  |  |  |  |
| M1: Intercept–only model | 3.893^***^ (.279^***^) |  |  | 66.914 | 26 | .809 | .845 | .075 [.053, .097] |  |  |  |  |  |
| M2: Linear model | 4.014^***^ (.300^***^) | $-$.050^***^ (.006) |  | 42.332 | 23 | .910 | .917 | .055 [.027, .080] |  | M2–M1 | 16.834 (3)^***^ | -0.101 | 0.020 |
| M3: Free–change model | 3.953^***^ (.297^***^) | $-$.037^**^ (.004) |  | 22.724 | 18 | .978 | .974 | .031 [.000, .065] |  | M3–M1 | 17.969 (5)^**^ | -0.068 | 0.024 |

*Note*. *M*= mean; σ^2^= variance; χ^2^= chi-square; *df*= degrees of freedom; CFI= Comparative Fit Index; TLI= Tucker–Lewis Index; RMSEA= Root Mean Square Error of Approximation and 90% confidence interval; Δ= change in parameter. The row highlighted in gray indicates the selected model following the comparison.

^**^ *p* < .01 ^***^ *p* < .001

# **Table S8.**

# Multigroup Latent Growth Curve analyses of intergroup solidarity

|  | Growth Factors | | | |
| --- | --- | --- | --- | --- |
|  | Native Italian Adolescents | | Migrant Adolescents | |
| Statuses | Intercept *M* (σ^2^) | Slope *M* (σ^2^) | Intercept *M* (σ^2^) | |
|  |  |  |  |  |
| Achievement | ${4.25}_{a}^{***}$ (.164^**^) | ${-.049}_{a}^{***}$ (.004^*^) | ${4.15}_{a}^{***}$ (.325^***^) | |
| Early Closure | ${3.94}_{b}^{***}$ (.269^***^) | ${-.039}_{a}^{***}$ (.003^**^) | ${3.86}_{ab}^{***}$ (.271^***^) | |
| Moratorium | ${3.98}_{b}^{***}$ (.328^***^) | ${-.041}_{a}^{***}$ (.006^**^) | ${4.06}_{a}^{***}$ (.226^***^) | |
| Searching Moratorium | ${4.04}_{b}^{***}$ (.207^**^) | ${-.044}_{a}^{***}$ (.003^*^) | ${4.01}_{ab}^{***}$ (.249^***^) | |
| Diffusion | ${3.65}_{c}^{***}$ (.206^***^) | ${-.047}_{a}^{*}$ (.014^*^) | ${3.61}_{bc}^{***}$ (.199) | |

*Note*. *M*= mean; σ^2^= variance.

Subscript letters within columns indicate significant coefficient differences, as emerged from the Wald test pairwise comparisons.

^*^ *p* < .05; ^**^ *p* < .01 ^***^ *p* < .001

# **Figure S1.**

# Estimated growth of intergroup solidarity of native Italian adolescents in different educational identity statuses

**
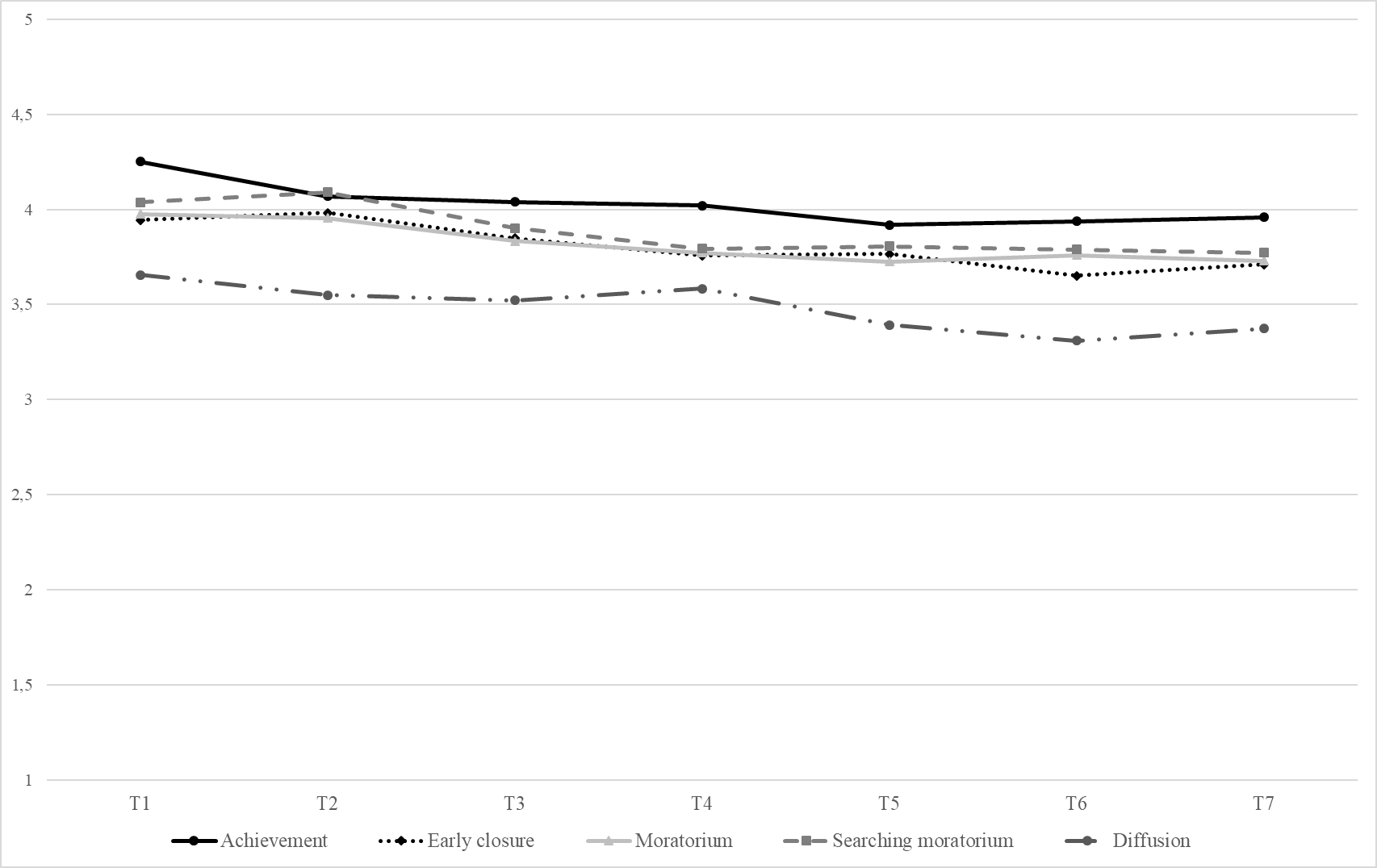
**

# **Figure S2.**

# Estimated growth of intergroup solidarity of migrant adolescents in different educational identity statuses

**
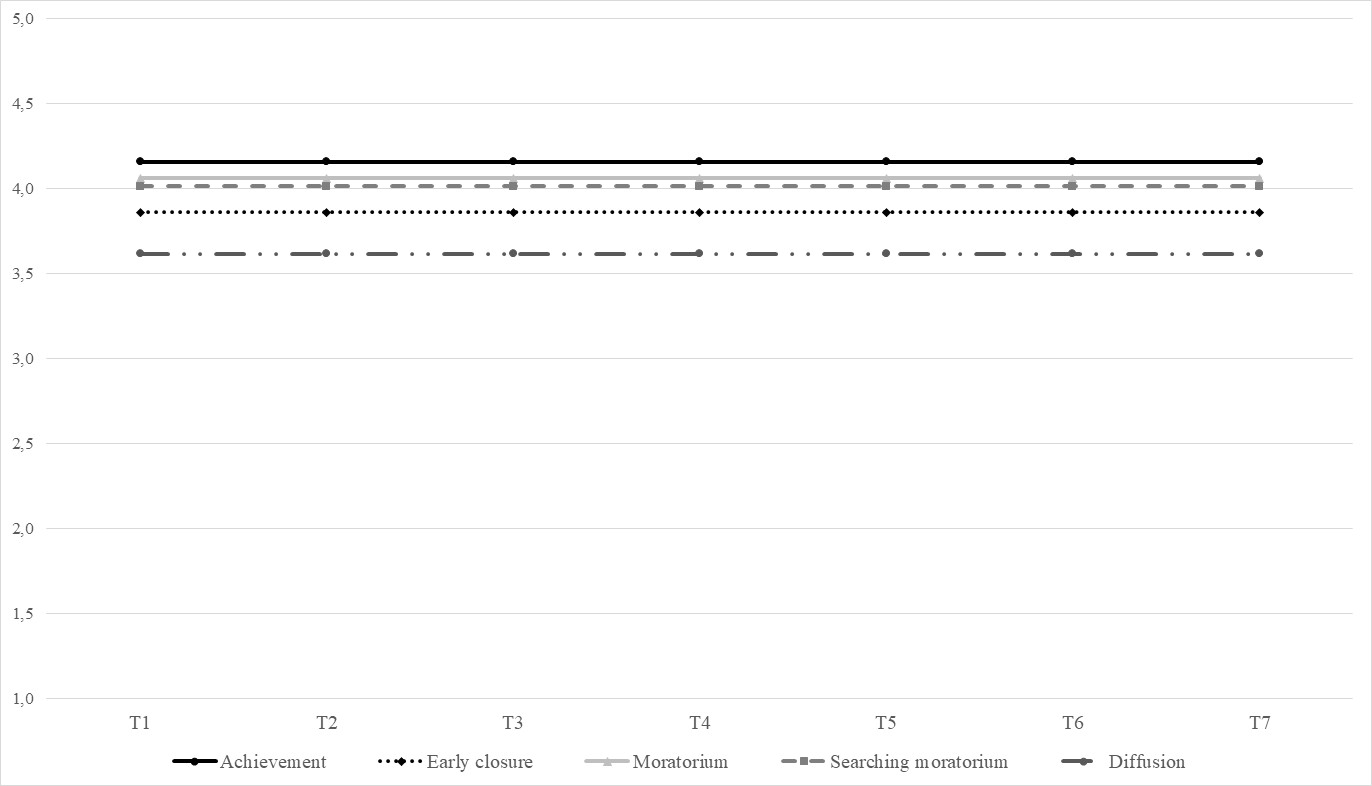
**
